# Human monocyte subsets differ in their capacity to form extracellular traps

**DOI:** 10.1038/s41420-024-02034-y

**Published:** 2024-06-12

**Authors:** Nahla Ibrahim, Viktoria Knöbl, Hubert Hayden, Wolfgang M. Bauer, Nina Worel, Christoph Neumayer, Christine Brostjan

**Affiliations:** 1 Division of Vascular Surgery, Department of General Surgery, Medical University of Vienna and University Hospital Vienna, Vienna, Austria; 2 Department of Dermatology, Medical University of Vienna and University Hospital Vienna, Vienna, Austria; 3 Department of Transfusion Medicine and Cell Therapy, Medical University of Vienna and University Hospital Vienna, Vienna, Austria

**Keywords:** Immune cell death, Monocytes and macrophages

Extracellular traps (ETs), particularly those produced by neutrophils (NETs), have emerged as vital components of innate immunity: Decondensed chromatin strands are released to entrap pathogens in a web-like structure of DNA and cellular proteins that assist in killing the immobilized organisms [1]. In chronic, non-infectious conditions, ETs have been linked to autoimmunity, cardiovascular disease, and cancer [2, 3]. While much attention has been given to NETs, Granger et al. reported that human blood monocytes are also able to release ETs in response to various stimuli and the composition of these monocyte extracellular traps (MoETs) was remarkably similar to expelled DNA-protein complexes of NETs [4]. Notably, the heterogeneous monocyte population can be divided into 3 distinct subsets based on their expression of the co-receptor for toll-like receptor 4 (CD14) and the Fcɣ receptor III (CD16) and these subpopulations differ in their predominant functions: Classical monocytes (CD14++/CD16-) are highly effective phagocytes and represent the largest fraction (80–90% of circulating monocytes), while intermediate (CD14++/CD16+) and non-classical (CD14+/CD16++) monocytes comprise about 2-10% and primarily engage in tissue remodeling or vessel patrolling, respectively [5–7]. To address the question of whether these populations also differ in their capacity to generate extracellular traps, our study has characterized human MoET dynamics of isolated classical, intermediate, and non-classical monocyte subsets in vitro.

The release of monocyte DNA was assessed by Sytox Green incorporation in a time course experiment (detailed methods in Supplemental Material). Two stimuli in low, medium, or high dose were chosen for distinct pathways of ET induction, i.e. activation of protein arginine deiminase 4 by the calcium ionophore A23187 or NADPH oxidase 2 activation by PMA. Untreated total monocytes remained unaffected over 300 min, while A23187-stimulated monocytes released DNA in a concentration-dependent manner starting at 105 min (Fig. 1A and Supplementary Fig. S1A). PMA activation was not able to substantially stimulate monocytes to release DNA even at high levels of 125 nM (Fig. S1B). Thus, further experiments were focused on A23187 treatment at the non-saturating dose of 2.5 μM.

**Fig. 1:**
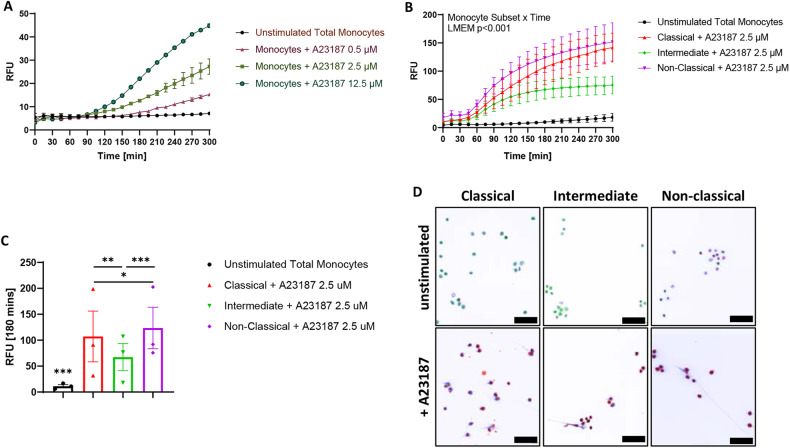
DNA release of monocyte subsets after stimulation by A23187. Extracellular DNA was assessed in monocytes by incorporation of Sytox Green dye and measurement of relative fluorescence units (RFU) over 5 h. **A** DNA release by total (unfractionated) monocytes in response to stimulation by the calcium ionophore A23187 (0.5 μM–2.5 μM–12.5 μM). **B** Time course of DNA release by FACsorted, A23187 (2.5 µM) activated monocyte subsets including linear mixed effects model (LMEM) analysis of the four test groups. **C** Group comparison at 180 min: *p < 0.05, **p < 0.01, ***p < 0.001 (LMEM monocyte subset x ordinal time). Data of 3 independent experiments (based on distinct donors) are presented as mean ± SEM. **D** Monocyte subsets were labeled with FITC for CD14 (green) and VioBlue for CD16 (red) for FACsorting, then seeded on coverslips with or without stimulation by A23187 for 90 min at 37 °C before fixation and DNA staining with DRAQ5 (blue). Overlay images of triple stainings are shown on a white background to facilitate DNA strand visualization. Scale bar: 50 µm.

To set DNA release by monocytes in relation to the well-described ET formation by neutrophils, the same number of neutrophils (isolated from three different donors) was either left untreated or stimulated with the commonly established NET induction doses of 1 μM A23187 or 1 nM PMA. Neutrophils from all three healthy donors started DNA release after 30 min of A23187 stimulation and plateaued around 90 min (Fig. S1C). Comparably, extracellular DNA was detected as early as 75 min post-PMA stimulation and plateaued at 120 min (Fig. S1D). Yet, untreated neutrophils had a higher NET auto-activation than monocytes over the 5-hour time frame.

The major monocyte subsets were then isolated by fluorescence-activated cell sorting (FACsorting) to a purity ranging between 80-100% in three experiments based on distinct blood cell donors (Fig. S2). DNA release upon A23187 stimulation of the distinct monocyte subsets was detected via Sytox Green incorporation over a 5-hour time course. Non-classical and classical monocytes showed high levels of extracellular DNA, while intermediate monocytes displayed only moderate DNA release (Fig. 1B). Analysis by linear mixed effects model (LMEM) for the interaction of monocyte subsets over time reached a significance level of *p* < 0.001 for combined (Fig. 1B) or pairwise analyses (Supplementary Table S1). The pairwise comparison of activated monocyte subsets at ordinal time points further revealed that DNA release by non-classical and intermediate monocytes started to diverge after 75 min of A23187 stimulation (*p* = 0.023), whereas classical and intermediate monocytes separated at 165 min (*p* = 0.030). The classical and non-classical subsets differed significantly after 75 min (*p* = 0.011) and re-converged after 210 min. Thus, differences between the three populations were best reflected at 180 min of MoET induction by calcium ionophores (Fig. 1C).

Isolated, labeled monocyte subsets were also seeded on glass coverslips, and DNA staining was performed to visualize MoET formation after 90 min of A23187 stimulation (Fig. 1D and Fig. S3). Each subset was microscopically confirmed by the antibody labels used for FACsorting. Untreated classical monocytes were positive for CD14 and negative for CD16, while stimulated classical monocytes substantially reduced their CD14 signal and turned highly positive for CD16. MoET formation was confirmed by the detection of extracellular DNA strands. Comparably, untreated intermediate monocytes were positive for CD14 and showed moderate levels of CD16. After A23187 stimulation, the intermediate monocyte CD14 signal was greatly diminished while their CD16 signal was remarkably increased. Extracellular DNA strands extending from the cells confirmed the ability of intermediate monocytes to form MoETs. In line with their flow cytometric profile, untreated non-classical monocytes had a weak CD14 signal but were positive for CD16. Post-A23187 activation, the non-classical monocytes released DNA strands and further intensified their CD16 expression.

Setting our data in relation to previous findings, Granger et al. [4] observed that A23187 at 5 µM was more potent in triggering MoET formation than PMA at 25 nM, but reported a comparable DNA release pattern for monocytes and neutrophils. In contrast, we found A23187 to trigger DNA release from monocytes in a concentration-dependent manner while PMA exhibited a poor dose-response which differed from neutrophil ability to efficiently expel DNA in response to both stimuli. While this may relate to distinct modalities of monocyte/neutrophil isolation or donor differences between our studies, it should be emphasized that PMA is known to induce differentiation rather than activation of monocytes which could also explain the lack of DNA release following PMA treatment [8]. In line, another study examining the release of MoETs in response to fungal or bacterial stimuli mentioned that monocytes were unable to undergo ET release in response to PMA or lipopolysaccharide [9]. These authors also gathered the first evidence that distinct monocyte subsets have the potential for MoET formation: In confocal microscopy of total monocytes exposed to *Candida albicans*, Halder et al. detected expelled DNA strands from both CD16+ (non-classical and/or intermediate) and CD16- (classical) monocytes [9]. Our study takes these findings a step further by differentiating between classical, intermediate, and non-classical monocytes and by quantifying DNA release of FACsorted monocyte subsets in a time course. The comparison of sorted subsets may be of particular importance since our microscopic analysis revealed a rapid loss of surface CD14 and concomitant increase in CD16 on stimulated monocytes. While this is a known response to short-term activation [10] rather than differentiation from classical to intermediate and non-classical monocytes that require about 24 h [11], it interferes with subset identification in total monocyte populations post-stimulation.

Our observation that classical and non-classical monocytes exhibit a substantially stronger MoET capacity than intermediate monocytes is remarkable since the differentiation of monocytes proceeds in a continuum from classical to intermediate to non-classical monocytes [11]. This points to a distinct relevance of MoET formation for the functions attributed to the subsets: While pathogen immobilization by MoETs may assist in vessel patrolling and phagocytic activities of non-classical and classical monocytes, intermediate monocyte functions in tissue remodelling and antigen presentation do not necessarily involve MoET formation. Our study thus supports the notion that monocyte subsets exert distinct contributions to acute or chronic conditions by MoET formation in line with their known roles in pathogen defense, tissue homeostasis, and regeneration.

### Supplementary information

Supplemental Material

## Data Availability

The raw data supporting the conclusions of this article will be made available by the authors upon request, without undue reservation.
