# Supplementary material for: Human monocyte subsets differ in their capacity to form extracellular traps
Source: Cell Death Discov. 2024 Jun 12;10:281. doi: 10.1038/s41420-024-02034-y (PMC11169614; doi:10.1038/s41420-024-02034-y)
Supplement: Supplementary file 1 — Supplemental Material [file 41420_2024_2034_MOESM1_ESM.pdf]

Correspondence – Supplemental Material

Human monocyte subsets differ in their capacity to form extracellular traps

Nahla Ibrahim[1]*, Viktoria Knöbl[1]*, Hubert Hayden[1], Wolfgang M. Bauer[2], Nina Worel[3], Christoph Neumayer[1] and Christine Brostjan[1#]

[1]*Division of Vascular Surgery, Department of General Surgery, Medical University of Vienna and University Hospital Vienna, Vienna, Austria*

[2]*Department of Dermatology, Medical University of Vienna and University Hospital Vienna, Vienna, Austria*

[3]*Department of Transfusion Medicine and Cell Therapy, Medical University of Vienna and University Hospital Vienna, Vienna, Austria*

*These authors contributed equally to this work.

[#]Address for correspondence: Christine Brostjan
Division of Vascular Surgery, Department of General Surgery
Medical University of Vienna and University Hospital Vienna
Anna Spiegel Center for Translational Research 25.05.002
Währinger Gürtel 18-20, 1090 Vienna, Austria
T: + 43 1 40400 73514 / F: + 43 1 40400 73593
E: christine.brostjan@meduniwien.ac.at

SUPPLEMENTARY MATERIALS AND METHODS

Monocyte isolation

As sorting of monocyte subsets required a high number of total monocytes, their isolation was based on leukoreduction system chambers (LRSCs) which represent a byproduct of platelet collection with the Spectra OPTIA® device (Terumo BCT) and are an abundant source of peripheral blood mononuclear cells (PBMCs). LRSCs were provided by the Dept. of Transfusion Medicine and Cell Therapy (Medical University of Vienna) as approved by the local ethics committee (no. 1711/2020). Informed consent was obtained from blood donors. For the first enrichment, 7 mL of cell suspension retrieved from LRSCs were diluted with 20 ml phosphate-buffered saline (PBS) before density gradient centrifugation at 1200 x g for 10 min using 11 ml Ficoll-Paque PLUS (Cytiva, Uppsala, Sweden) and SepMate™-50 IVD tubes (STEMCELL Technologies Germany GmbH, Cologne, Germany). The retrieved PBMCs were centrifuged once more with washing buffer, i.e. with PBS containing 2% fetal bovine serum (FBS, Linaris Biological Products GmbH, Dossenheim, Germany) and 2 mM ethylenediaminetetraacetic acid (EDTA, Merck KGaA, Darmstadt, Germany). Monocytes were further enriched by negative selection using the EasySep™ Human Monocyte Enrichment Kit without CD16 Depletion (STEMCELLTechnologies, Germany GmbH) following the manufacturer's protocol which resulted in monocyte enrichment from 30-35% to 90-95%.

Monocyte subset sorting

Per $1x10^6$ monocytes, 2 µl REAlease® CD14-FITC (130-112-074, Miltenyi Biotec B.V. & Co. KG, Bergisch Gladbach, Germany), 2 µl REAlease® CD16-VioBlue (130-120-878, Miltenyi Biotec B.V. & Co. KG), 2 µl REAlease® CD56-PE releasable antibodies (130-117-731, Miltenyi Biotec B.V. & Co. KG), and 2.5 µl 7-AAD (A07704, Beckman Coulter GmbH, Vienna, Austria) were added. Monocytes were subjected to fluorescence-activated cell sorting (FACsorting) into their respective subsets according to their expression of CD14 and CD16 **(Fig. S2)**, using a BD FACSAria™ III Cell Sorter (BD Austria GmbH, Vienna, Austria) and the BD FACSDiva™ Software (BD FACSDiva™ Software v9.0, BD Austria GmbH). Cells were collected in 1 ml heat-inactivated FBS, centrifuged at 300 x g for 10 min and subsequently resuspended in 980 µl washing buffer. To regain CD14/CD16 accessibility and obtain unlabeled sorted monocyte subsets for ET induction, the REAlease® antibodies were detached from the cell surface by adding 20 µl of release buffer (130-120-675, Miltenyi Biotec B.V. & Co. KG). Of note, the temperature of cell suspensions was kept at 4°C for all incubation and centrifugation steps of monocyte isolation and sorting, to avoid artificial cell activation and differentiation.

Of note, monocyte purification from leukoreduction system chambers (by using a negative selection kit and FACsorting) entailed that monocyte and neutrophil isolations were based on distinct

blood donors. However, it greatly reduced monocyte contamination by granulocytes. As the more commonly used HLA-DR antibodies in monocyte identification by flow cytometry were not available in REAlease® format, priority was given to CD56 antibodies to efficiently discriminate between non-classical monocytes and contaminating NK cells in subset purification **(Fig. S2)**.

Neutrophil isolation

Human neutrophils were isolated from EDTA anti-coagulated blood at room temperature using Histopaque density gradient centrifugation (#10771, #11191, Sigma-Aldrich, St. Louise, MO, USA) at 700 x g for 30 min without brakes. The neutrophil layer was aspirated and washed with PBS without calcium and magnesium (800 x g for 5 minutes at 4°C) followed by hypotonic lysis of red blood cells and re-establishment of isotonicity.

DNA release assay

After antibody removal, $1x10^5$ monocytes in 100 µl of Hank's balanced salt solution with calcium and magnesium ($HBSS^{++}$) were seeded in a black, clear-bottom 96-well plate and then stimulated with 0.5 µM, 2.5 µM, or 12.5 µM of the calcium ionophore A23187 (Sigma-Aldrich, St. Louis, MO, USA) or with 5 nM, 25 nM, 125 nM of phorbol 12-myristate 13-acetate (PMA, Sigma-Aldrich) for 300 min at 37 °C. To quantify the DNA release, 5 µM of SYTOX Green (Thermo Fisher Scientific Inc., Waltham, MA, USA) was added and fluorescence was measured at 15 min intervals at excitation/emission of 485/520 nm using the VarioSkan Lux plate reader (Thermo Fisher Scientific Inc).

For comparison with NETs, neutrophils isolated by density gradient centrifugation from three different healthy donors were seeded ($1x10^5$) and left untreated or stimulated with 1 µM A23187 or 1 nM PMA, and DNA release was quantified as described for monocytes.

Immunofluorescence microscopy

For the staining of MoETs, about $5x10^4$ cells (200 µl of sorted, CD14-FITC and CD16-VioBlue labeled monocyte subsets in $HBSS^{++}$) were seeded on glass coverslips in a 24 well-plate and pre-incubated for 30 min at 37 °C to allow for cell adhesion. Monocytes were then either left untreated or stimulated with 12.5 µM A23187 for 90 min at 37 °C to induce MoET formation. Cells were subsequently fixed with 4% paraformaldehyde for 10 min at room temperature then washed 2x with PBS. DRAQ5 ™ nucleic acid stain (ab108410, Abcam, Cambridge, UK) was applied at 1:1000 dilution in PBS for 20 min. Coverslips were again washed and finally mounted with Fluoromount G® (Southern Biotech, Birmingham, AL, USA). Fluorescence images (4-6 regions of interest per coverslip) were acquired using an automated Axio Observer Z1 microscope (Carl Zeiss MicroImaging, Inc., Oberkochen, Germany) with a 20x objective and the TissueFAXS scan software (TissueGnostics GmbH, Vienna, Austria).

Statistical analysis

Time course data were plotted in a line graph with mean ± SEM, and the difference in DNA release (expressed in relative fluorescence units, RFU) among monocyte subsets was calculated by a linear mixed effects model (LMEM) with "time" as the main effect, "monocyte subset x time" as fixed effect, and "time" as the random, multifactorial effect (with compound symmetry covariance structure). For the time point of 180 min a bar chart (mean ± SEM) is given with individual data points representing the separate experiments. All analyses were conducted with GraphPad Prism version 9.0.0 for Windows (GraphPad Software, San Diego, CA, USA) and SPSS 27.0 software (IBM, Armonk, NY, USA) and a significance level of $p < 0.05$ was applied.

SUPPLEMENTARY TABLE

Supplementary Table S1: Pairwise comparison of A23187 (2.5 µM) activated monocyte subsets by LMEM as summarized in table format for estimates of fixed effects with 95% confidence intervals (CI) in reference to Fig. 1B.

LMEM Monocyte Subset x Time	Estimate	P-value	Lower 95% CI	Upper 95% CI	Residual
**Classical vs Intermediate**	0.145	p<0.001	0.123	0.167	119
**Classical vs Non-Classical**	-0.068	p<0.001	-0.095	-0.042	176
**Intermediate vs Non-Classical**	-0.213	p<0.001	-0.244	-0.182	233

SUPPLEMENTARY FIGURES

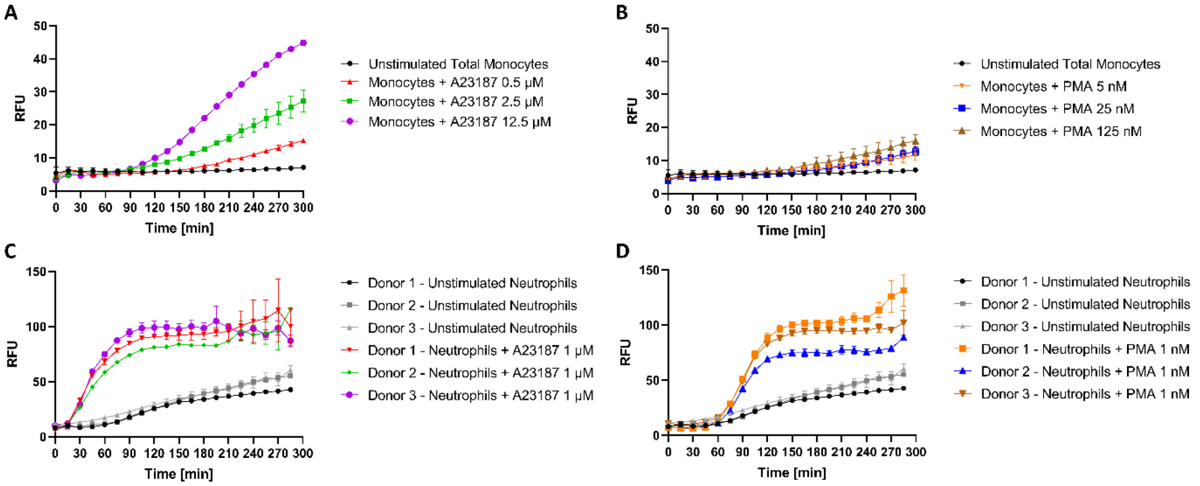

Supplementary Figure S1: Time course of DNA release by myeloid cells upon A23187 or PMA stimulation. Extracellular DNA was assessed in (unfractionated, total) monocytes or neutrophils by incorporation of Sytox Green dye and measurement of relative fluorescence units (RFU) over 5 hours. (A) Total monocyte DNA release in response to stimulation by the calcium ionophore A23187 (0.5 µM – 2.5 µM – 12.5 µM). (B) Total monocyte response to PMA stimulation (5 nM – 25 nM – 125 nM). Of note, monocyte experiments were repeated with a second blood donor. In comparison, neutrophils from three different donors were tested for DNA release after stimulation with (C) A23187 and (D) PMA at the established NET-inducing concentrations (1 µM and 1 nM, respectively). Data is presented as mean ± SEM of technical replicates.

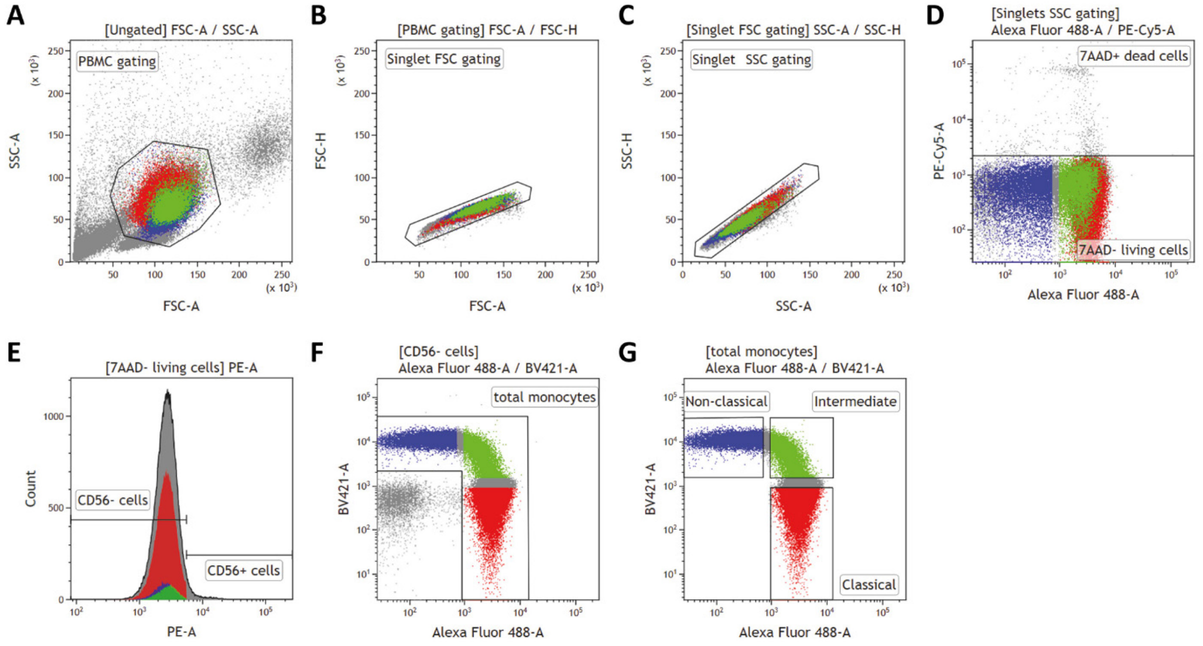

Supplementary Figure S2: Gating strategy for FACsorting of monocyte subsets. Representative flow cytometry plots showing (A) forward scatter (FSC) and side scatter (SSC) based pre-gating of PBMCs, (B) single cell discrimination based on FSC area versus height signal, (C) single cell discrimination based on SSC area versus height signal, (D) exclusion of dead cells based on 7-AAD incorporation (PE-Cy5 channel), (E) exclusion of natural killer cells based on CD56 staining (PE channel), (F) exclusion of remaining non-monocytes based on immunofluorescence signals for CD14 (Alexa Fluor 488 channel) and CD16 (BV421 channel), and (G) monocyte subset gating according to CD14/CD16 intensity for sorting into classical (CD14++/CD16-), intermediate (CD14++/CD16+) and non-classical (CD14+/CD16++) monocytes.

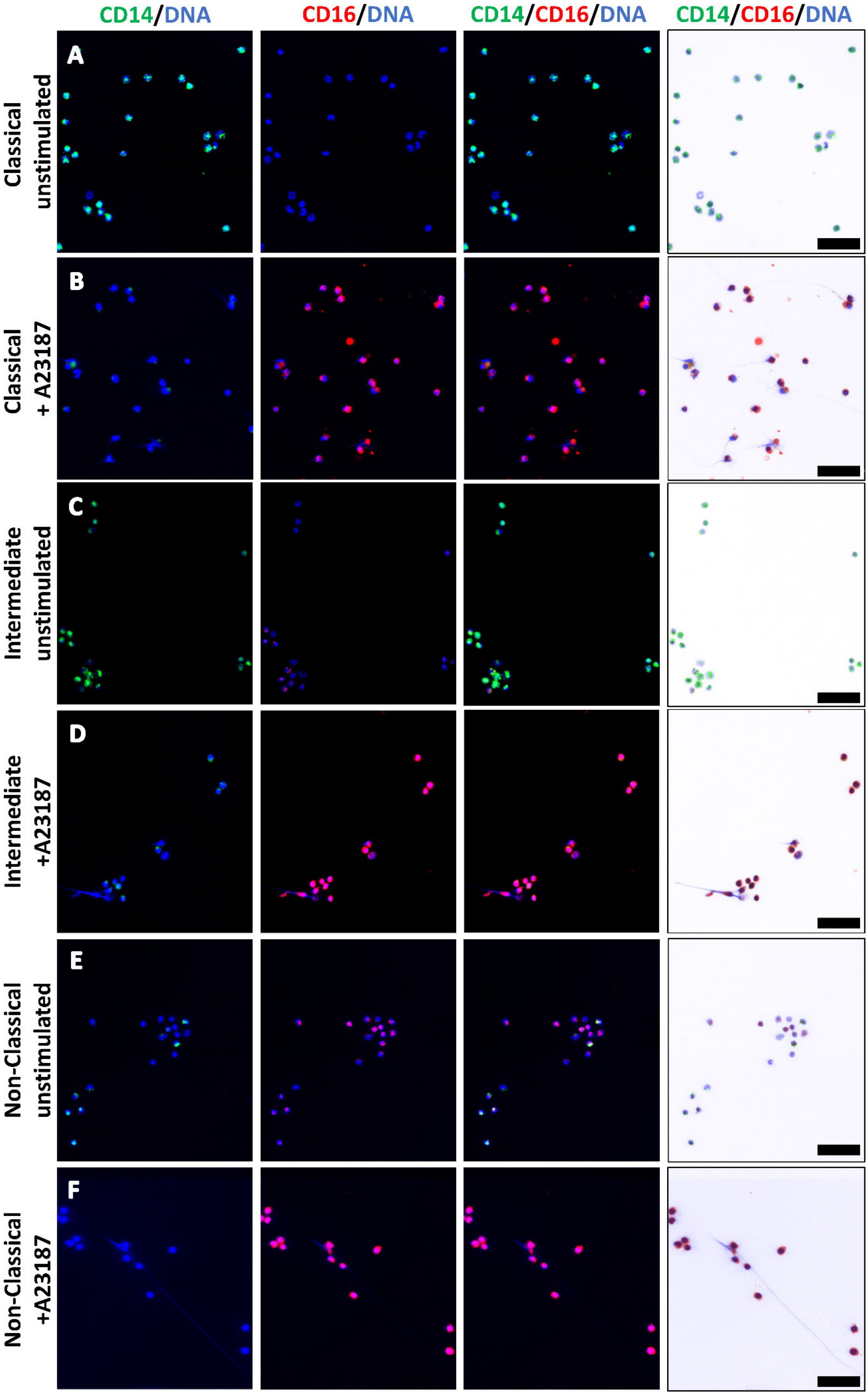
CD14/DNA
CD16/DNA
CD14/CD16/DNA
CD14/CD16/DNA
A
B
C
D
E
F
Classical unstimulated
Classical + A23187
Intermediate unstimulated
Intermediate +A23187
Non-Classical unstimulated
Non-Classical +A23187

Supplementary Figure S3: MoET formation by A23187-activated monocyte subsets. Monocyte subsets were labeled with FITC for CD14 (green) and VioBlue for CD16 (red) for FACsorting, then seeded on coverslips with or without stimulation by A23187 for 90 min at 37 °C before fixation and DNA staining with DRAQ5 (blue): (A) untreated classical monocytes, (B) stimulated classical monocytes, (C) untreated intermediate monocytes, (D) stimulated intermediate monocytes, (E) untreated non-classical monocytes, and (F) stimulated non-classical monocytes. Overlay images of triple staining were additionally visualized on a light background to facilitate DNA strand visualization. Scale bar: 50 µm.
